# Effect of green tea extract on bonding durability of an etch-and-rinse adhesive system to caries-affected dentin

**DOI:** 10.1590/1678-775720150518

**Published:** 2016

**Authors:** Carolina CARVALHO, Fernando Pelegrim FERNANDES, Valeria da Penha FREITAS, Fabiana Mantovani Gomes FRANÇA, Roberta Tarkany BASTING, Cecilia Pedroso TURSSI, Flávia Lucisano Botelho AMARAL

**Affiliations:** Faculdade de Odontologia e Centro de Pesquisas Odontológicas São Leopoldo Mandic, Campinas, SP, Brasil.

**Keywords:** Chlorhexidine, Camellia sinensis, Matrix metalloproteinases, Tensile strength

## Abstract

**Objective:**

Green tea extract has been advocated as a matrix metalloproteinase (MMP) inhibitor; however, its effect on bond durability to caries-affected dentin has never been reported. Thus, the aim of this *in vitro* study was to evaluate the effect of two MMP inhibitors (2% chlorhexidine and 2% green tea extract), applied after acid etching, on bond durability of an etch-and-rinse adhesive system to caries-affected dentin.

**Material and Methods:**

Occlusal enamel was removed from third molars to expose the dentin surface, and the molars were submitted to a caries induction protocol for 15 days. After removal of infected dentin, specimens were conditioned with 37% phosphoric acid (15 seconds) and randomly divided into three groups, according to the type of dentin pretreatment (n=10): NT: no treatment; GT: 2% green tea extract; CLX: 2% chlorhexidine. The etch-and-rinse adhesive system (Adper™ Single Bond 2, 3M ESPE, St. Paul, MN, USA) was applied according to the manufacturer's instructions, and composite resin restorations were built on the dentin. After 24 hours, at 37°C, the resin-tooth blocks were sectioned perpendicularly to the adhesive interface in the form of sticks (0.8 mm^2^ of adhesive area) and randomly subdivided into two groups according to when they were to be submitted to microtensile bond strength (μTBS) testing: immediately or 6 months after storage in distilled water. Data were reported in MPa and submitted to two-way ANOVA for completely randomized blocks, followed by Tukey’s test (α=0.05).

**Results:**

After 24 hours, there was no significant difference in the μTBS of the groups. After 6 months, the GT group had significantly higher μTBS values.

**Conclusion:**

It was concluded that the application of 2% green tea extract was able to increase bond durability of the etch-and-rinse system to dentin. Neither the application of chlorhexidine nor non-treatment (NT - control) had any effect on bond strength after water storage.

## INTRODUCTION

The evolution of adhesive restorative materials and the knowledge of caries lesion progression have led to the development of the minimally invasive Dentistry concept, whose principles advocate minimum intervention in cavitated carious lesions and maximum preservation of sound dental structure^26^. This interventionist philosophy separates carious dentin on two levels: the outer layer or infected dentin^10^, which must be removed, and the inner layer of dentin, with a lower level of infection, known as caries-affected dentin, which can be remineralized and must be preserved^3^ ^,^ ^26^. Caries-affected dentin has greater porosity, partial demineralization and altered ultrastructural collagen^28^. These modifications lead to the formation of a more irregular hybrid layer, with greater area of exposed collagen^18^ ^,^ ^28^ and lower bond strength, as compared to sound dentin^9^ ^,^ ^29^.

In addition, the adhesive interface is susceptible to hydrolytic degradation, expressed by an increase in the caries-affected dentin^9^. This degradation occurs in stages^18^: water is absorbed by the polymer when hydrolytic degradation first begins; resin components are leached from the hybrid and/or adhesive layer; the exposed collagen fibrils are degraded by endopeptidases present in the dentin matrix (MMPs)^17^ and/or by cysteine cathepsins^27^. MMPs are secreted as inactive proenzymes, but promote the degradation of collagen fibril, elastin and extracellular matrix components when activated. It is known that these enzymes can be activated at a lower pH – as occurs when dentin is conditioned by acid etching – but function the best in a neutral pH^13^ – as occurs following application of the etch-and-rinse adhesive system. The collagenolytic function of MMPs is also relevant in caries-affected dentin tissue, since they are activated at a low pH, during bacteria lactic fermentation and subsequent neutralization by dentin buffer systems^24^.

The understanding of how the mechanism of action of MMPs and cysteine cathepsins influences collagen degradation has encouraged the development and evaluation of substances that may inhibit their function. Among these substances, chlorhexidine has been promoted as a synthetic inhibitor of MMPs, since it does not interfere with the immediate bonding to the dental substrate^3^ ^,^ ^12^ ^,^ ^16^ and increases the longevity of adhesive restorations^4^ ^,^ ^11^. Green tea, on the other hand, has been described as a natural inhibitor of MMPs^7^. It is made from *Camellia sinensis* and is composed of polyphenols named catechins, such as epicatechin (EC), epigallocatechin (EGC), epicatechin gallate (ECG) and epigallocatechin gallate (EGCG), pointing out that ECG and EGCG are strongly related to MMP inhibition^7^. Results from a previous study^16^ revealed that application of a green tea solution reduced immediate bond strength to dentin, but kept it stable in the long-term. However, the effect of green tea extract application on the bond durability of an etch-and-rinse adhesive to caries-affected dentin has not yet been assessed.

Considering the relevance of studying bond strength to caries-affected dentin, and seeking to find alternative approaches in the context of retarding hybrid layer degradation, this study aimed at making an *in vitro* assessment of the effect of two metalloproteinase inhibitors, chlorhexidine and green tea, applied after acid etching, on bond durability of an etch-and-rinse adhesive system to caries-affected dentin, after six months of storage in water. The null hypothesis to be tested was that neither the MMP inhibitor solution nor the water storage period would have any effect on bond strength to caries-affected dentin.

## MATERIAL AND METHODS

### Ethical aspects

The present study was approved by the Local Research Ethics Committee (No. 2012/0200).

### Experimental design

This *in vitro* study involved a microbiological model of dentin caries induction, according to that proposed by Sanabe, et al.^18^ (2011). The factors under study were: 1. The solution applied to the dentin surface after acid conditioning: 2% green tea extract (GT) or 2% chlorhexidine digluconate solution (CLX), compared with the control group (no treatment – NT); 2. Time points of microtensile bond strength testing: after storage in water for 24 hours or for 6 months.

The quantitative outcome variable was the microtensile bond strength value, in MPa. The bond strength average of sticks from the same tooth became the value for that particular experimental unit. The failure pattern was described in terms of percentages.

**Figure 1:**
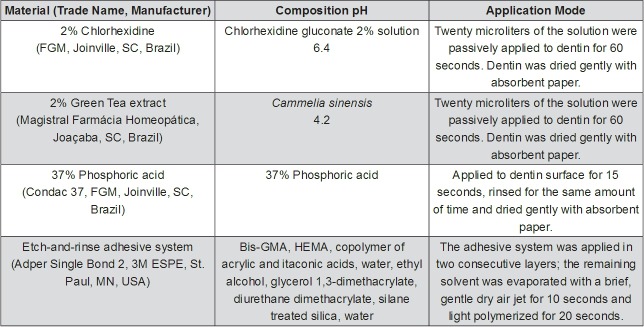
Main composition/pH and application modes of the materials used

The materials cited in the experimental design, as well as their composition and mode of use, are described in Figure 1.

### Dentin slab preparation

Thirty human third molars, extracted for reasons not related to those of the present research and stored in thymol (0.1%, pH 7.0) after extraction, were used in this experiment. The teeth were submitted to debriding with scalpel blades and periodontal curettes. Then, they were cross-sectioned using a water-cooled diamond saw (15 HC series, Buehler Ltd., Lake Bluff, IL, USA) in a sectioning machine (Isomet 1000 Precision Diamond Saw, Buehler Ltd., Lake Bluff, IL, USA), which removed the occlusal third from the crown to obtain a large dentin surface in the middle third, perpendicular to the long axis of the tooth. Dentin surfaces were flattened in a water-cooled polishing machine (Politriz Aropol 2V, Arotec, São Paulo, SP, Brazil) with decreasing granulations (#400, #600 and #1200) of water abrasive paper (Imperial Wetordry, 3M, Sumaré, SP, Brazil).

### Artificial caries induction protocol

The protocol for artificial caries induction was obtained from the study by Sanabe, et al.^18^ (2011). In brief, the tooth root apices were sealed with a microhybrid composite resin (Filtek Z250, 3M ESPE, St Paul, MN, USA) and the teeth were rendered waterproof with one layer of epoxy resin (Araldite, Ciba Especialidades Químicas, São Paulo, SP, Brazil) and one layer of an acid resistant varnish (nail polish, Colorama, CEIL, São Paulo, SP, Brazil), leaving only the dentin surface exposed. All the teeth were then autoclaved at 121°C for 20 min.

A 2% *Streptococcus mutans* strain ATCC 25175 (108 cfu/ml) (André Toselo Foundation Tropical Culture Collection) was inoculated into a pre-autoclaved cariogenic solution. The teeth were immerged in the cariogenic solution and incubated in microaerophilia for 14 days. During this period, the cariogenic solution was renewed every 48 hours, but without inoculation of new *S. mutans* strains. Autoclaving was repeated after the end of the incubation period. The biofilm was removed with gauze, and the insulating materials (varnish and epoxy resin) were removed with scalpel blades. The teeth were then rinsed with distilled and deionized water.

### Adhesive procedures

The infected dentin was removed with a #4 spherical steel bur at low speed, which was replaced after 5 times of use. The softened dentin tissue was removed until obtaining tissue that was harder and more resilient to the touch with a probe, without exerting pressure on the dentin. A single operator was calibrated to perform this procedure.

Dentin slabs were submitted to conditioning with 37% phosphoric acid for 15 seconds and rinsed with distilled water for the same amount of time. They were randomly divided into three groups, according to the solution to be applied to the dentin surface: 2% GT, 2% CLX, or NT (no solution applied, used as control). In brief, solutions of twenty microliters of 2% GT or of 2% CLX were passively applied to dentin for 60 seconds. The dentin was dried gently with absorbent paper. The etch-and-rinse adhesive system was then applied to the dentin fragments, which were restored with a microhybrid resin composite (Filtek Z250, 3M ESPE, St. Paul, MN, USA). The final resin-dentin blocks were 4-mm high. Light polymerization was performed on both the adhesive system (Adper™ Single Bond 2, 3M ESPE, St. Paul, MN, USA) and the microhybrid composite, for the time recommended by the manufacturers (Figure 1), using a visible light-curing unit (Ultralux EL, Dabi Atlante, Ribeirão Preto, SP, Brazil). The output of the light-curing unit was measured periodically with a radiometer (Newdent Equipamentos Ltda., Ribeirão Preto, SP, Brazil), and a minimal range of 630 mW/cm^2^ was observed.

The resin-dentin blocks were kept in relative humidity at 37°C for 24 hours and sectioned perpendicularly to the bonding surface into 0.9-mm thick slabs, using a water-cooled diamond saw. Multiple beam-shaped sticks were obtained by rotating samples 90° and sectioning them again lengthwise, each with a cross-sectional surface area of 0.8 mm^2^. Half of the sticks from the same resin-dentin block were submitted to µTBS testing after 24 hours. The other half was kept in distilled water, which was changed every two days, and kept in a bacteriological oven for 6 months.

### Microtensile bond strength (µTBS) Testing

The specimens were attached to a µTBS testing device with cyanoacrylate adhesive (Super Bonder Gel, Henkel Ltda., Itapevi, SP, Brazil). They were subjected to tensile stress in a universal testing machine at a crosshead speed of 0.5 mm/min and a 50 N load cell until fracture. The bond strength values were expressed in kgf/cm^2^ and converted to MPa after measuring of the cross-sectional area at the fracture site with a digital caliper (Mitutoyo Corp, Tokyo, Japan). The comparison was conducted using the average value of each tooth (n=10).

After bond strength testing, the failure pattern of each stick was analyzed under a stereomicroscope (EK3ST, CQA, São Paulo, SP, Brazil) at 30x magnification to assess the failure modes, which were classified as adhesive (lack of adhesion), cohesive in dentin (failure of the dental substrate), cohesive in composite resin (failure of the resin composite) or mixed (adhesive and cohesive failures).

### Statistical analysis

Based on the normal distribution of the data, two-way analysis of variance (ANOVA) for completely randomized blocks and Tukey’s test were applied. Statistical calculations were performed with SPSS 20 (SPSS Inc., Chicago, IL, USA). The significance level was set at 5%. The failure pattern was described with descriptive statistics (percentage terms).

## RESULTS

The two-way ANOVA for randomized blocks showed that there was significant interaction between the specific treatments and the time periods (p=0.003). Tukey’s test indicated that there was no significant difference in bond strength between the groups treated with green tea and with chlorhexidine, as compared to the control group after 24 hours (Table 1). In contrast, the group to which green tea extract was applied yielded significantly higher bond strength values after 6 months (Table 1). Moreover, there was no significant difference in bond strength values after 6 months of storage, except for the group to which green tea was applied, which showed significant increase in strength, in relation to the 24-hour period.

**Table 1:** Mean (standard deviation) of the experimental groups

Time	Dentin Pretreatment		
	Absent (control)	Green Tea	Chlorhexidine
24 hours	24.3 (8.6)^Aa^	23.0 (6.3)^Ab^	23.3 (6.0)^Aa^
6 months	21.6 (6.4)^Aa^	35.7 (8.4)^Ba^	23.0 (7.2)^Aa^

Means followed by the same letters (uppercase letters within each row and lowercase letters within each column) are not significantly different (α=0.05)

**Figure 2:**
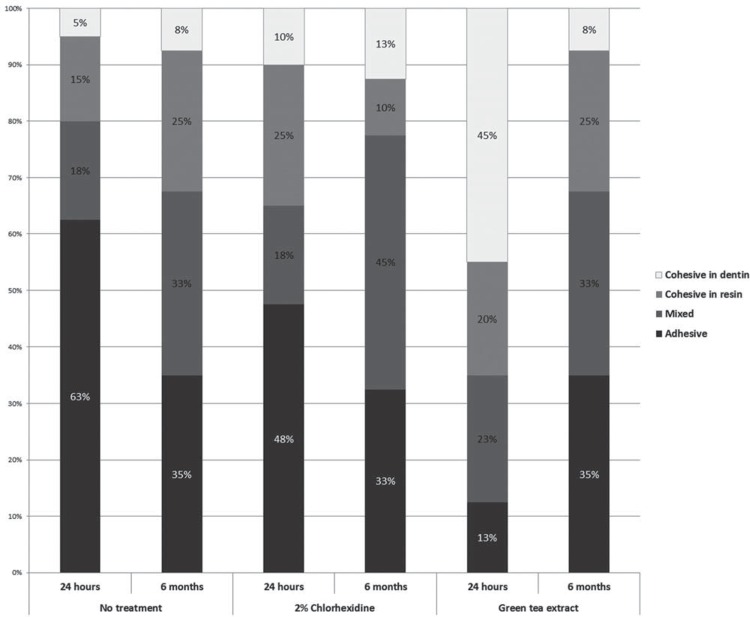
Failure patterns for each experimental group, in percentage terms

As for the failure mode (Figure 2), it was observed that, after 24 hours of storage in water, the group that received no treatment (control) presented a predominance of adhesive failure (63%), followed by mixed (18%), cohesive in resin composite (15%) and cohesive in dentin (5%) failures. In comparison, after 6 months of storage, most of the failures in the same group (control) were of the adhesive type (35%), followed by mixed failures (33%), failure of the resin composite (25%) and cohesive failure in dentin (8%).

Most of the failures in the group to which chlorhexidine digluconate was applied were adhesive (48%), followed by cohesive in resin composite (25%), mixed (18%) and cohesive in dentin (10%), at the 24-hour time point, whereas, after 6 months of water storage, most of the failures in the same group were mixed (45%), followed by adhesive (33%), cohesive in dentin (13%), and cohesive in resin composite (10%).

Most of the failures in the group to which green tea was applied were cohesive in dentin (45%), followed by mixed (23%), cohesive in resin composite (20%), and adhesive (13%), at the 24-hour time point, whereas, after 6 months, most of the failures in the same group were adhesive (35%), followed by mixed (33%), cohesive in resin composite (25%), and cohesive in dentin (8%).

## DISCUSSION

The knowledge that dentin matrix metalloproteinases and cysteine cathepsins are involved in the hybrid layer degradation process has driven the search for substances that inhibit the action of these enzymes^27^. Among them, the most prominent substance is chlorhexidine digluconate, a synthetic inhibitor of MMPs and cysteine cathepsins^25^.

Most of the studies that assess the degradation of the hybrid layer and the role of the MMPs in this process involve performing adhesive system procedures on sound dentin^2^ ^,^ ^4^ ^,^ ^11^ ^,^ ^20^ ^,^ ^22^ ^,^ ^30^. However, the hybrid layer formed on caries-affected dentin (CAD) is more irregular and has greater collagen exposure^18^ ^,^ ^28^, with reduced bond strength, in comparison with sound dentin^9^ ^,^ ^29^. Moreover, hydrolytic degradation is more pronounced in the CAD adhesive interface^9^, where MMPs and cysteine cathepsins exert substantial activity^27^.

Thus, this study aimed at assessing the effect of both synthetic (2% chlorhexidine) and natural (2% green tea extract) metalloproteinase inhibitor solutions, when applied after acid conditioning, on either immediate or long-term bond strength of an etch-and-rinse adhesive system to caries-affected dentin. The null hypothesis was rejected, since the results of this research have shown that 2% green tea extract (natural inhibitor of metalloproteinases) was able to increase long-term bond strength significantly. On the other hand, the application of 2% chlorhexidine (synthetic inhibitor of metalloproteinases) was able to maintain the long-term bond strength on a level equal to that of the control group.

Studies have shown that 2% chlorhexidine used as a therapeutic dentin pretreatment did not interfere with immediate bond strength^2^ ^,^ ^4^ ^,^ ^14^ ^,^ ^16^ ^,^ ^20^, corroborating the results of this study. A recent meta-analytic review^15^ has shown that 2% chlorhexidine does not affect the immediate bond strength to dentin and improves the bond after 6 months in water storage. However, the control group (no treatment) in the present study also presented bond stability over time; that is, the control and the chlorhexidine groups presented statistically similar results. It is hypothesized that chlorhexidine substantivity does not exceed 8 weeks^3^ or 3 months^21^, and that the specimens in stick form may have facilitated the dissolution of the chlorhexidine solution, therefore curbing a more beneficial effect. Nonetheless, when analyzing the control group, in which no bond strength alteration was observed after 6 months, other considerations should be borne in mind. First, all the teeth were autoclaved, as a measure to promote microbiological caries induction^18^. It can be speculated that high temperatures may affect proteinases by denaturing them. Sanabe, et al.^18^ (2011) stated that MMP-8 is more fragile in resisting high temperatures, whereas gelatinases such as MMP-2 are more resistant. Although autoclaving may reduce enzymatic activity, the study by these authors^18^ showed that aging in water for 6 months resulted in degradation of the resin-dentin bond, and that this degradation decelerated in the presence of chlorhexidine. Another important hypothesis is related to the researched storage time of 6 months. According to De Munck, et al.^6^ (2012), the simple water storage of specimens has a clear bond-degrading effect. However, the literature makes no definitive conclusion in regard to the minimal period of water storage that promotes degradation in the hybrid layer and a consequent decrease in bond strength. By applying an adhesive system under simulated intrapulpal pressure and storing specimens for two years in artificial saliva, Mobarak, et al.^14^ (2011) observed that the bond strength to caries-affected dentin was similar between 2% chlorhexidine-treated and non-treated dentin. However, at a concentration of 5%, chlorhexidine was able to prevent loss in bond strength after 2 years. Another point is related to water as the storage medium. Based on the conclusion that MMPs require zinc and calcium to trigger activity, Tezvergil-Mutluay, et al.^23^ (2010) reported that using water as a storage medium underestimates the hydrolytic activity of endogenous dentin MMPs, because it promoted the loss of calcium and zinc ions from dentin matrices, rather than restoring them. In this context, the use of solutions that simulate oral fluids and longer storage periods should be researched in future studies.

The most surprising result of this research is that the 2% green tea extract increased the etch-and-rinse adhesive system bond strength. Bond stability has been reported using 0.05% green tea extract in 9 months of water storage^30^. The application of diluted green tea obtained from commercially sold tea was found to be prejudicial to the immediate bond strength of the etch-and-rinse adhesive to human dentin; however, after 6 months of storage in water, the bond remained stable, without showing any detrimental or beneficial effects^16^. Industrialized green tea has many other compounds associated with it, and this may have contributed to the initial, unfavorable results in the study by Monteiro, et al.^16^ (2013). In fact, it has been proposed that the application of a solution containing the main catechin in green tea - epigallocatechin gallate (EGCG) - in pretreatment form^19^ or incorporated into the etch-and-rinse adhesive system^8^ promotes significant bonding stability to dentin, compared with the control group (no treatment), as well as increased bond strength to bleached enamel, due to the antioxidant properties also exhibited by EGCG. Although the studies reporting the effects of EGCG application on dentin MMP inhibition are scant, it has been reported that EGCG significantly enhances both the pro- and active-MMP-2 binding to TIMP-2, the natural MMP inhibitor physiologically present in dentin^5^.

Moreover, because the studies evaluating green tea extract and EGCG solutions on dentin are scarce, and because most studies have been conducted on sound dentin, the comparison of results has been rendered difficult. It is speculated that green tea extract could have increased the bond strength of the etch-and-rinse adhesive system because it was tested on caries-affected dentin, where the occurrence of MMPs and cysteine cathepsins is aggravated^27^. Additionally, because green tea polyphenol contains EGCG, it has been classified as a proanthocyanidin (PAC), a highly hydroxylated structure capable of forming an insoluble complex with proteins^1^. For this reason, green tea polyphenol has been advocated as a natural collagen cross-linking agent, able to enhance the mechanical properties of dentin and to reduce the dentin biodegradability of host-derived MMPs^1^. In fact, cohesive in dentin failures were more prevalent (45%) at the 24-hour period of water storage than at the 6-month period (8%).

As for the failure mode of the experimental groups, there was a predominance of adhesive and mixed failures. Monteiro, et al.^16^ (2013) noted that their reported failures did not seem to follow any pattern according to treatment type, and highlighted the high frequency of adhesive and mixed failures, not only in the control group but also in the 2% chlorhexidine and the green tea groups. Mobarak, et al.^14^ (2011) observed that there was a predominance of adhesive and mixed failure modes when using 2% and 5% chlorhexidine as a pretreatment when the specimens were tested for bond strength after 24 hours of storage. After testing the specimens for bond strength after 6 months of water storage under simulated pulpal pressure, Campos, et al.^2^ (2007) observed that most of the failure modes belonged to the adhesive type when using 2% chlorhexidine as a pretreatment.

Considering the above, it is suggested that further studies should be conducted using green tea extract as a metalloproteinase inhibitory and bioactive substance, not only in performing mechanical tests, as in this study, but also in order to ascertain the inhibitory effect of green tea extract on the presence and activity of dentin MMPs.

## CONCLUSION

It was concluded that the application of a green tea extract solution was able to increase the long-term bond strength to caries-affected dentin. Neither the application of chlorhexidine nor non-treatment (NT - control) had any effect on the bond strength after water storage.
